# The preventive effects of ondansetron on chemotherapy-induced nausea and vomiting in adult cancer patients: systematic review from ClinicalTrials.gov

**DOI:** 10.3389/fphar.2023.1310455

**Published:** 2023-11-23

**Authors:** Ahmed M. Ashour

**Affiliations:** Pharmacology and Toxicology Department, College of Pharmacy, Umm Al-Qura University, Makkah, Saudi Arabia

**Keywords:** clinical trials, nausea, vomiting, ondansetron, patients

## Abstract

**Purpose:** Cancer is a neoplastic transformation that affects tissue. Among the many complications associated with cancer treatment, managing the distressing side effects of chemotherapy-induced nausea and vomiting (CINV) is of main concern. Ondansetron is a selective serotonin 5-HT3 receptor antagonist that has emerged as an essential medication against CINV in adult cancer patients. Ondansetron efficacy and tolerability have made it a primary medication in CINV prophylaxis and treatment regimens. The study aims to offer a detailed overview of ondansetron’s effectiveness, safety, and impact on patients’ lives, ultimately contributing to the ongoing research to enhance the quality of cancer care.

**Methods:** On 4 September 2023, a search was conducted of the ClinicalTrials.gov database using the search terms “cancer,” “ondansetron,” and “Zofran.” Inclusion and exclusion criteria were defined to select relevant clinical trials. Included trials were completed with results and interventional studies that assessed the preventive effects of ondansetron on CINV in adult cancer patients.

**Results:** A total of 23 clinical trials were identified, with only 13 of them focusing on investigating the preventive effects of ondansetron on CINV in adult cancer patients. The collective findings from these trials showed an effective management of CINV using ondansetron.

**Conclusion:** Through a comprehensive overview of clinical trials, the use of ondansetron in adult cancer patients represents a significant improvement in CINV management.

## Introduction

Cancer, a complicated disease characterized by abnormal cell growth and proliferation, causes a significant health challenge. According to the World Cancer Report 2020, cancer is ranked as the second highest global cause of mortality. In 2018, it was estimated that cancer accounted for 9.6 million deaths (Sung et al., 2021). In addition, cancer was classified as being responsible for the second highest death rate in the United States by the American Cancer Society, second only to heart disease (Siegel et al., 2023). Anti-neoplastic drugs such as alkylating, antimetabolic, and antimitotic agents, are one of the most important cancer treatments but they are associated with the complication of chemotherapy-induced nausea and vomiting (CINV) (Hoofnagle, 2013). CINV represents a distressing consequence of cancer chemotherapy, significantly compromising patients’ wellbeing and treatment adherence (Roila et al., 2016). CINV not only disrupts daily life but also discourages patients from continuing essential cancer therapies (Supportive and Board, 2023). Currently, medications including neurokinin-1 receptor antagonists, glucocorticoids, butyryl benzenes, phenothiazines, benzamides, antihistamines, and anticholinergics have been used for the prevention of CINV. However, they have a short-acting duration and limited effect on post-treatment vomiting and anti-nausea (Weibel et al., 2021). Recent guidelines recommend serotonin-3 receptor (5-HT3) antagonists as the first-line drugs for the prevention and treatment of CINV (Kovac, 2016).

Many reports have examined the effectiveness of ondansetron as a preventive measure against CINV, focusing on its mechanisms of action and clinical outcomes (Rao and Faso, 2012; Theriot et al., 2018). Ondansetron targets specific receptors of the gastrointestinal tract and central nervous system (Griddine and Bush, 2022). As ondansetron blocks serotonin receptors, especially 5-HT3 receptors, it disrupts the signaling pathways of the vomiting center and the chemoreceptor trigger zone (CTZ) (Theriot et al., 2018). Chemotherapy patients experience decreased nausea and vomiting as a result (Rao and Faso, 2012). Ondansetron’s mechanism of action has proven highly effective when used in combination with chemotherapy (Griddine and Bush, 2022). In adults with cancer, ondansetron has been shown to prevent CINV in many clinical studies (Ye et al., 2001; Rao and Faso, 2012; Theriot et al., 2018; Griddine and Bush, 2022). A significant decrease and delay in acute CINV was reported among patients receiving ondansetron-based antiemetic therapy (Rao and Faso, 2012). These findings not only improved the wellbeing of cancer patients but also maintained adherence to chemotherapy regimens, thereby maximizing the effectiveness of therapeutic. Ondansetron’s safety profile and well-tolerated nature make it a necessary medication in the comprehensive care of adult cancer patients undergoing chemotherapy (Rao and Faso, 2012).

Primarily, this paper aims to deliver a comprehensive summary of the preventive effects of ondansetron on CINV in adult cancer patients. The study is focused specifically on evaluating the clinical results and consequences associated with the administration of ondansetron, analyzing, and investigating the database of clinical data and research. This systematic review contributes to CINV management and assists healthcare experts and researchers in making informed decisions regarding antiemetic therapy for cancer patients. Thus, this paper seeks to highlight the significance of ondansetron as a key medication in improving the overall wellbeing and treatment experience of adult cancer patients undergoing chemotherapy.

## Material and methods

### Search strategy

On 4 September 2023, this study conducted an extensive search on ClinicalTrials.gov, a well-known online database globally recognized for its comprehensive collection of clinical trials conducted in 221 countries. This invaluable resource serves as a publicly accessible registry, thoroughly archiving medical studies involving human volunteers, thus providing a great source of information for this research. The analysis was centered on finding the application of ondansetron in contexts related to cancer. Specifically focused on entries linked to pharmacological studies, to extract relevant data from this extensive repository. Following the search, 23 clinical trials were identified to be associated with the use of ondansetron in cancer-related scenarios. To ensure the precision and relevance of this review, a careful screening process was started. This process involved excluding trials addressing unrelated medical conditions, incomplete studies without conclusive outcomes, and observational clinical trials. After this detailed screening, only 13 clinical trials met the determined eligibility criteria for our review. These criteria included a primary research emphasis on cancer, the administration of ondansetron (commonly referred to as Zofran) as a therapeutic intervention, the completion of the studies, and the inclusion of adult patients aged between 18 and 64 years. As a result, data from these 13 clinical trials were considered for inclusion in our analysis, forming the foundational basis upon which findings are analyzed.

### Data extraction

The collection of data for the study was a thorough process involving both manual extraction and the retrieval of information from ClinicalTrials.gov. This database served as a primary source of this research. To begin, we carefully retrieved data related to interventional clinical trials, providing us with insights into the specific interventions being studied. Our focus remained on medical conditions, with “cancer” emerging as the primary disease of interest. At the same time, we focused on “ondansetron” as the targeted medical treatment, allowing us to examine its effectiveness in cancer-related contexts across various clinical trial phases. Moreover, we ensured the inclusivity of all clinical trial phases, providing a complete view of ondansetron’s applicability in cancer treatment. We carefully collected critical data points such as the trial outcomes, providing insights into the effectiveness of ondansetron; the number of participants involved, enabling us to measure the sample sizes and statistical significance; the years in which these trials were conducted, revealing potential trends and developments over time; and the specific conditions under examination, clarifying the context within which ondansetron was being evaluated. The inclusion of participant counts, represented as “n,” offered a quantitative dimension to our analysis, aiding in the assessment of the trials’ scale and statistical strength. This comprehensive and systematic approach to data collection from ClinicalTrials.gov forms the main source of our research.

## Results

As of 4 September 2023, a total of 23 clinical trials were investigated of ondansetron’s treating CINV. Among these trials, 13 trials were exclusively to examine the effects of ondansetron on CINV following chemotherapy. It is essential to highlight that these 13 studies had completed their research phases, ensuring a strong dataset for our analysis. Detailed information, including their titles, current statuses, targeted medical conditions, administered interventions, outcome measures, and the sizes of the study (Table 1), facilitates a comprehensive understanding of the research. The findings from these conducted trials have consistently highlighted the substantial benefits of integrating ondansetron into the management of CINV. Ondansetron has consistently demonstrated remarkable efficacy in alleviating both the delayed and acute phases of vomiting and nausea that are frequently triggered by chemotherapy regimens. Moreover, it is noteworthy that ondansetron is often employed synergistically with other antiemetic medications, thereby providing patients with a comprehensive strategy for effectively controlling the distressing symptoms associated with CINV. This research not only supports the significance of ondansetron in enhancing the quality of life for cancer patients but also contributes to the understanding of the ondansetron effects of CINV.

**TABLE 1:** (Data from https://clinicaltrials.gov, updated on 04-09–2023).

Study title	Conditions	Interventions	Study phase	Outcome measures	Number of participants	Year of the study
“Oral Ondansetron Versus Transdermal Granisetron (Sancuso) for Women With Cervical, Endometrial or Vaginal Cancer Receiving Pelvic Chemoradiation” ^(15)^	Cervical cancer	Ondansetron	Phase 3	Percentage of Participating patients with a response rate to anti-emetic therapy days 4–7 of each chemotherapy cycle	76	2019
				Percentage of Participating patients with a response rate to anti-emetic therapy at 0–24 h after each chemotherapy cycle		
“Dexamethasone and Ondansetron Hydrochloride or Palonosetron Hydrochloride in Preventing Nausea and Vomiting in Patients Receiving Doxorubicin Hydrochloride and Cyclophosphamide for Early-Stage Breast Cancer” ^(16)^	Male breast cancer	Ondansetron	Not applicable	Count of patients achieving complete response	41	2017
				Number of days patients experienced emetic episodes and received rescue medication		
				Number of participating patients who experienced emesis within 48 h of receiving chemotherapy		
“Ondansetron Versus Palonosetron Antiemetic Regimen Prior to Highly Emetogenic Chemotherapy (HEC)” ^(17)^	Malignant neoplasm	Ondansetron	Not applicable	Overall complete response (CR), defined as no emesis and no rescue medication, following the initial course of HEC	40	2011
“Ondansetron Plus Dexamethasone With or Without Metoclopramide as Antiemetic Prophylaxis After Receiving Cisplatin” ^(18)^	Cancer	Ondansetron	Not applicable	Number of patients who had a complete response Toxicities and severity of nausea and vomiting	162	2010
“Aprepitant in Preventing Nausea and Vomiting in Patients Who Are Undergoing a Stem Cell Transplant” ^(19)^	Cancer	Ondansetron	Not applicable	Number of patients who remained emesis-free throughout the study period	40	2009
				Safety in transplant population Changes to appetite and taste Effect on nausea		
“Antiemetic Therapy With or Without Olanzapine in Preventing Chemotherapy-Induced Nausea and Vomiting in Patients with Cancer Receiving Highly Emetogenic Chemotherapy” ^(20)^	Hematopoietic/lymphoid cancer	Ondansetron	Phase 3	The proportion of patients with no nausea	401	2015
	Unspecified adult solid tumor					
“Palonosetron Versus Ondansetron for the Prevention of Nausea and Vomiting” ^(21)^	Acute myeloid leukemia	Ondansetron	Phase 2	Number of patients with complete response (CR)	150	2009
“Ondansetron in Preventing Nausea and Vomiting in Patients Undergoing Stem Cell Transplant” ^(22)^	Accelerated phase chronic myeloid leukemia (CML)	Ondansetron	Phase 2	Reduction in the rate of vomiting or nausea following ondansetron (when compared to historical FHCRC rates)	49	2009
	Adult acute lymphoblastic leukemia (ALL) in remission Adult acute myeloid leukemia (AML) in remission					
	Adult AML with 11q23 (MLL gene) abnormalities					
“Ondansetron Versus Aprepitant Plus Ondansetron for Emesis” ^(23)^	Hematological diseases	Ondansetron	Phase 2	Participant responses	122	2013
	Acute myeloid leukemia (AML)					
	Myelodysplastic syndrome (MDS)			Treatment success rate		
	Chronic myeloid leukemia (CML)					
“A Korean Study of Efficacy and Safety of Aprepitant-based Triple Regimen for the Prevention of Chemotherapy-induced Nausea and Vomiting in the First Cycle of Moderately Emetogenic Chemotherapy (Non-doxorubicin Hydrochloride [Adriamycin] and Cyclophosphamide Regimens)” ^(24)^	Nausea	Ondansetron	Phase 4	Percentage of patients with no vomiting—overall stage	494	2014
				Percentage of patients with a complete response (CR) –overall, delayed, and acute stages		
				Total number of emetic events—overall stage		
	Vomiting			Percentage of patients with no significant nausea and no vomiting—overall stage		
				Percentage of patients having no impact on their day-to-day life—overall stage		
				Number of patients not needing to use a rescue therapy—overall, delayed and acute stages		
				Percentage of patients who experienced one or more clinical adverse event		
				Percentage of patients with no vomiting—acute and delayed stages		
“Emend and Ondansetron Compared to Ondansetron Alone to Prevent CINV in Glioma Patients Receiving Temozolomide” ^(25)^	Nausea Vomiting	Ondansetron	Phase 2	The proportion of patients achieving complete control	136	2017
	Glioma			The proportion of patients experiencing acute and delayed complete response (CR)		
				Patient’s global satisfaction with the antiemetic regimen		
“A Study of IV Casopitant for the Prevention of Chemotherapy Induced Nausea and Vomiting” ^(26)^	Chemotherapy-induced nausea and vomiting	Ondansetron	Phase 3	Maximum nausea score, evaluated using a visual analog scale (VAS)	710	2009
				Percentage of patients receiving rescue medication		
				Percentage of patients who retched and/or vomited		
				Percentage of patients reporting significant nausea, defined as a maximum score that is ≥ 25 mm on the VAS		
				Percentage of patients reporting nausea, defined as a maximum score that is ≥ 5 mm on the VAS		
				Percentage of patients who experienced complete		
				protection, defined as complete responders		
“Aprepitant’s Effect on Drug Metabolism in Multi-Day Combination (CHOP/R-CHOP) Chemotherapy Regimen in Lymphoma Patients” ^(27)^	Lymphoma	Ondansetron	Not applicable	Ondansetron helped to prevent nausea and/or vomiting in patients with Non-Hodgkin’s Lymphoma	23	2011

## Discussion

From a total of 23 clinical trials, only 13 of them specifically focused on investigating the preventive effects of ondansetron on CINV in adults with cancer. The collective findings from these trials confirmed that ondansetron is effective in managing CINV. This evidence showed that ondansetron successfully prevents and alleviates the distressing side effects of vomiting and nausea in adults with cancer who are undergoing chemotherapy.

Ondansetron is a selective serotonin receptor antagonist used for the pharmacotherapy of CINV, which is a significant challenge to cancer patients and may weaken treatment compliance and overall wellbeing (Ye et al., 2001). Ondansetron’s mechanism of action involves blocking serotonin receptors in the brain and gastrointestinal tract, modifying the emetic signals triggered by chemotherapy (Rao and Faso, 2012). This pharmacological agent has gained importance due to its well-established efficacy in managing CINV, offering an essential treatment to alleviate the distressing side effects experienced by cancer patients undergoing chemotherapy (Rao and Faso, 2012).

Clinical studies have consistently demonstrated the effectiveness of ondansetron in reducing both acute and delayed phases of CINV (Rao and Faso, 2012). Its recommended dosage typically involves oral or intravenous administration, with varying regimens based on the chemotherapy emetogenicity and the patient’s specific risk factors (Rao and Faso, 2012). Ondansetron’s efficacy has been well documented in different cancer types and chemotherapy regimens, positioning it as a useful and reliable antiemetic agent (Griddine and Bush, 2022). The drug’s ability to provide substantial relief from nausea and vomiting provides the patient with comfort and facilitates adherence to treatment plans, thereby contributing to better therapeutic outcomes (Griddine and Bush, 2022).

While ondansetron is highly effective in managing CINV, its safety profile is another critical part of its pharmacotherapy. Clinical trials and real-world experience have generally shown that ondansetron is well tolerated, with a low incidence of severe adverse effects (Rao and Faso, 2012; Griddine and Bush, 2022). Commonly described side effects include constipation, headache, and transitory alterations to enzyme levels in the liver, which are typically mild (Smith, 1989). This beneficial safety profile enhances ondansetron’s in cancer treatment protocols, as it ensures that the benefits of CINV control do not affect patients or heightened risk**.** Therefore, the pharmacotherapy of ondansetron in cancer treatment has promise for further refinement and exploration.

The research identified patients that may benefit from ondansetron, considering factors such as age, gender, and cancer type. Ondansetron has also been shown to have synergies with other antiemetic agents, which warrants further investigation to optimize CINV management in the future. Ondansetron has long been known for its efficacy in reducing CINV, which confirms its role as a main therapeutic tool in many cancer patients and enhances the clinical efficacy and patient care in the field of oncology (Hewitt et al., 1993).

## Conclusion

The findings from the review suggest that the use of ondansetron for CINV after chemotherapy can be considered as a first-line option. The number of trials for ondansetron is extremely limited yet it can provide patients with a better quality of life. While acknowledging the relatively limited number of trials available for ondansetron, the collective evidence underscores its critical role in enhancing the quality of life for cancer patients undergoing chemotherapy. Ondansetron’s demonstrated efficacy in reducing CINV, with its well-tolerated safety profile, places it as a vital component of current cancer care strategies. This systematic review not only confirms the clinical importance of ondansetron but also highlights the pressing need for further research to expand our understanding of its potential applications, with a focus on improving treatment protocols and maximizing patient outcomes.

## Data Availability

The original contributions presented in the study are included in the article/Supplementary material, further inquiries can be directed to the corresponding author.

## References

[B1] Ashour A. M. (2023). Efficacy and safety of ondansetron for morning sickness in pregnancy: a systematic review of clinical trials. Front. Pharmacol. 14, 1291235. 10.3389/fphar.2023.1291235 37936910 PMC10625999

[B2] Griddine A. Bush J. S. (2022). “Ondansetron,” in StatPearls (Florida, United States: StatPearls Publishing).

[B3] Hewitt M. McQuade B. Stevens R. (1993). The efficacy and safety of ondansetron in the prophylaxis of cancer-chemotherapy induced nausea and vomiting in children. *Clin. Oncol*. R. Coll. Radiol. 5 (1), 11–14. ‏. 10.1016/s0936-6555(05)80686-7 8424909

[B4] Hoofnagle J. H. (2013). “LiverTox: a website on drug-induced liver injury,” in Drug-induced liver disease (Massachusetts, United States: Academic Press), 725–732.

[B5] Kovac A. L. (2016). Comparative pharmacology and guide to the use of the serotonin 5-HT3 receptor antagonists for postoperative nausea and vomiting. Drugs 76 (18), 1719–1735. ‏ 10.1007/s40265-016-0663-3 27988869

[B6] National library of medicine. (2009a). Ondansetron in preventing nausea and vomiting in patients undergoing stem cell transplant. NCT00795769. Available at: https://clinicaltrials.gov/study/NCT00795769

[B7] National library of medicine. (2009b). Palonosetron versus ondansetron for the prevention of nausea and vomiting. NCT01031498. Available at: https://clinicaltrials.gov/study/NCT01031498

[B8] National library of medicine. (2009c). Aprepitant in preventing nausea and vomiting in patients who are undergoing a stem cell transplant. NCT00248547. Available at: https://clinicaltrials.gov/study/NCT00248547

[B9] National library of medicine. (2009d). A study of IV casopitant for the prevention of chemotherapy induced nausea and vomiting, NCT00601172. Available at: https://clinicaltrials.gov/study/NCT00601172

[B10] National library of medicine. (2010). Ondansetron plus dexamethasone with or without metoclopramide as antiemetic prophylaxis after receiving cisplatin. NCT01093690. Available at: https://clinicaltrials.gov/study/NCT01093690

[B11] National library of medicine. (2011a). Ondansetron versus palonosetron antiemetic regimen prior to highly emetogenic chemotherapy(HEC), NCT01640340. Available at: https://clinicaltrials.gov/study/NCT01640340 10.1007/s00520-013-1865-9 PMC3769492 23748485

[B12] National library of medicine. (2011b). Aprepitant's effect on drug metabolism in multi-day combination (CHOP/​R-CHOP) chemotherapy regimen in lymphoma patients, NCT00651755. Available at: https://clinicaltrials.gov/study/NCT00651755

[B13] National library of medicine. (2013). Aprepitant versus ondansetron in preoperative triple-therapy treatment of nausea and vomiting, NCT01474915. Available at: https://clinicaltrials.gov/study/NCT01474915 10.1186/1745-6215-13-130 PMC3475143 22862827

[B14] National library of medicine. (2014). A Korean study of efficacy and safety of aprepitant-based triple regimen for the prevention of chemotherapy-induced nausea and vomiting in the first cycle of moderately emetogenic chemotherapy (Non-doxorubicin hydrochloride [adriamycin] and cyclophosphami. NCT01636947. Available at: https://clinicaltrials.gov/study/NCT01636947

[B15] National library of medicine. (2015). Antiemetic therapy with or without olanzapine in preventing chemotherapy-induced nausea and vomiting in patients with cancer receiving highly emetogenic chemotherapy. NCT02116530. Available at: https://clinicaltrials.gov/study/NCT02116530

[B16] National library of medicine. (2017a). Dexamethasone and ondansetron hydrochloride or palonosetron hydrochloride in preventing nausea and vomiting in patients receiving doxorubicin hydrochloride and cyclophosphamide for early stage breast cancer. NCT00343863. Available at: https://clinicaltrials.gov/study/NCT00343863

[B17] National library of medicine. (2017b). Emend and ondansetron compared to ondansetron alone to prevent CINV in glioma patients receiving temozolomide, NCT01450826. Available at: https://clinicaltrials.gov/study/NCT01450826

[B18] National library of medicine. (2019). Oral ondansetron versus transdermal granisetron (sancuso) for women with cervical, endometrial or vaginal cancer receiving pelvic chemoradiation, NCT01536392. Available at: https://clinicaltrials.gov/study/NCT01536392

[B19] Rao K. V. Faso A. (2012). Chemotherapy-induced nausea and vomiting: optimizing prevention and management. Am. Health and Drug Benefits 5 (4), 232–240. 24991322 PMC4046471

[B20] Roila F. Molassiotis A. Herrstedt J. Aapro M. Gralla R. J. Bruera E. (2016). 2016 MASCC and ESMO guideline update for the prevention of chemotherapy- and radiotherapy-induced nausea and vomiting and of nausea and vomiting in advanced cancer patients. Ann. Oncol. 27 (5), 119–133. 10.1093/annonc/mdw270 27664248

[B21] Siegel R. L. Miller K. D. Wagle N. S. Jemal A. (2023). Cancer statistics, 2023. CA Cancer J. Clin. 73 (1), 17–48. ‏. 10.3322/caac.21763 36633525

[B22] Smith R. N. (1989). Safety of ondansetron. Eur. J. Cancer Clin. Oncol. 25 (1), 47–54. 2533899

[B23] Sung H. Ferlay J. Siegel R. L. Laversanne M. Soerjomataram I. Jemal A. (2021). Global cancer statistics 2020: GLOBOCAN estimates of incidence and mortality worldwide for 36 cancers in 185 countries. CA Cancer J. Clin. 71 (3), 209–249. ‏. 10.3322/caac.21660 33538338

[B24] Supportive P. D. Q. Board P. C. E. (2023). “Nausea and vomiting related to cancer treatment (PDQ®),” in PDQ cancer information summaries (Bethesda, Maryland: National Cancer Institute US.

[B25] Theriot J. Wermuth H. R. Ashurst J. V. (2018). Antiemetic serotonin-5-HT3 receptor blockers. Florida, United States: StatPearls.

[B26] Weibel S. Schaefer M. S. Raj D. Rücker G. Pace N. L. Schlesinger T. (2021). Drugs for preventing postoperative nausea and vomiting in adults after general anaesthesia: an abridged Cochrane network meta-analysis. Anaesthesia 76 (7), 962–973. ‏ 10.1111/anae.15295 33170514

[B27] Ye J. H. Ponnudurai R. Schaefer R. (2001). Ondansetron: a selective 5-HT(3) receptor antagonist and its applications in CNS-related disorders. CNS Drug Rev. 7 (2), 199–213. ‏ 10.1111/j.1527-3458.2001.tb00195.x 11474424 PMC6741689

